# Impact of high fat and low vitamin A intervention on cortical transcriptional profiles in APP/PS1 mice and neuronal EEF1AKMT4-ECE2-associated amyloid metabolism

**DOI:** 10.3389/fnut.2026.1923538

**Published:** 2026-10-22

**Authors:** Xiuwen Ren, Lu Liu, Yiran Wang, Yu Liu, Xixiang Wang, Shaobo Zhou, Yunyun Gong, Yueyong Wang, Chen Miao, Ying Wang, Mengyang Sun, Linhong Yuan

**Affiliations:** 1 School of Public Health, Capital Medical University, Beijing, China; 2 Beijing Key Laboratory of Environment and Aging, Beijing, China; 3 Department of Epidemiology and Biostatistics, School of Public Health, Peking University, Beijing, China; 4 Institute of Reproductive and Child Health, School of Public Health, Peking University, Beijing, China; 5 School of Science, Faculty of Engineering and Science, University of Greenwich, Chatham, United Kingdom; 6 School of Food Science and Nutrition, University of Leeds, Leeds, United Kingdom; 7 Suzhou Research Center of Medical School, Suzhou Hospital, Affiliated Hospital of Medical School, Nanjing University, Suzhou, China

**Keywords:** Alzheimer's disease, EEF1AKMT4-ECE2, high fat diet, RNA-sequencing, vitamin A

## Abstract

**Background:**

Vitamin A (VA) deficiency and lipid metabolism disorders are associated with the pathogenesis of Alzheimer's disease (AD). However, their effects on cortical gene expression profile and the molecular pathways related to amyloid generation remain insufficiently understood.

**Methods:**

3-month-old APP/PS1 mice were fed with control, high fat, high fat with normal or low VA containing diets for 8 months. RNA-sequencing technique was applied to measure the cortical gene expression profile. Differentially expressed genes were further validated by real-time quantitative PCR and Western blot analysis. Additionally, SH-SY5Y neuronal cells were used to explore the functional role of the differential expressed molecule EEF1AKMT4-ECE2 (ECE2) in amyloid β (Aβ) metabolism.

**Results:**

Transcriptome analysis identified multiple differentially expressed genes related to diets containing different fat and/or VA, which are mainly involved in amyloid protein metabolism, neuronal differentiation, myelin structure, and metabolic signal transduction. Among them, *Ece2* gene was identified as a key nutrient-sensitive gene, showing a significant upregulated expression under high-fat diet intervention. *Hes3* and *Fam168b* were among the differentially expressed genes in the cortical of mice treated with diets contained different dosage of VA, and these genes potentially related to neuronal differentiation and myelin structure. In SH-SY5Y cells, Aβ treatment significantly up-regulated ECE2 protein expression. ECE2 activation rebalanced Aβ species toward Aβ_40_, yet its efficacy was undermined by high-fat and VA-deficient status, which triggered BACE1 over-expression, ultimately driving intracellular Aβ accumulation and extracellular Aβ_40_ elevation.

**Conclusion:**

High fat and low VA nutritional status significantly modified the transcriptional profile in the cortex of APP/PS1 mice. ECE2 is a nutrient-sensitive molecule involved in the regulation of cortical Aβ metabolism under high-fat and low-VA conditions.

## Graphical Abstract

**Figure:**
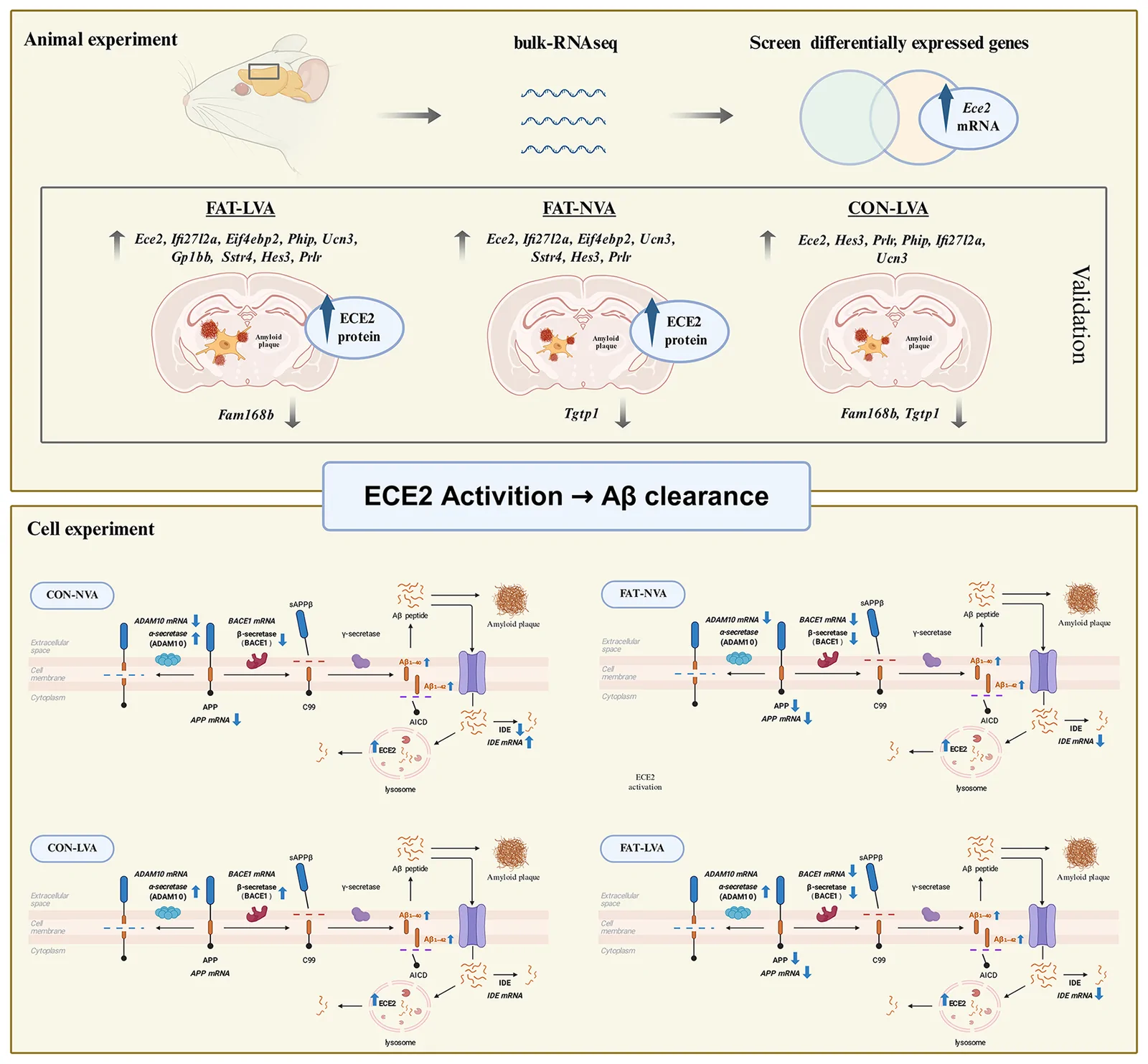

## Introduction

Alzheimer's disease (AD) is a neurodegenerative disease that accounts for 60% to 80% of dementia. It was reported that there would be 152 million AD patients worldwide by 2050 with the largest increase occurring in developing countries (1). The prevalence of dementia in population aged 60 years and above was 6.04%, and among which AD accounts for 3.94 %. It was estimated that there are about 9.83 million AD patients in China (2), and following population aging, this number will be further increased. Therefore, the prevention of AD has become an urgent public concern. The typical pathology of AD is characterized by the formation and accumulation of extracellular amyloid β (Aβ) in the brain parenchyma and cerebrovascular system, neurofibrillary plaques in neurons, and brain inflammatory changes caused by glial cell proliferation (3). Unfortunately, to date, there is still no efficient method available for clinical treatment of AD, therefore, identification of the risk factors and molecules involving in the abnormal Aβ metabolism in brain provide potential targets for the prevention of AD.

Vitamin A (VA), also named retinol, is a fat-soluble essential micronutrient, which has been proven to protect cell oxidative damage and regulate cell proliferation (4). Retinol was generally metabolizes to form retinoic acid (RA), and as the activity form of VA, RA could bind to its receptors- retinoic acid receptors (RAR) and retinol-like X receptors, to regulate multiple gene transcription and expression (5), including the genes correlated with cell apoptotic and antioxidant activities. VA was also reported to involve in maintaining the normal physiological function of brain, and are closely related to the division and differentiation of neuron, synaptic formation, neurite growth, as well as the early development of the brain. Maintaining an optimal VA nutritional status is commonly recommended to prevent cognition decline and AD (6). Clinical study has observed decreased blood VA levels in AD patients aged 50 and above, and low daily dietary VA intakes, suggesting the association of VA deficiency and the increased risk of AD (6). Others also reported that insufficient VA intake or dysfunction of RARs can lead to Aβ deposition, loss of synaptic plasticity, and memory impairment (7). VA deficiency has been found to cause spatial learning and memory abilities impairment, and promoted the progress of AD in rodents (8). Additionally, VA could act as an antioxidant to prevent the oxidative damage medicated by reactive oxygen species in neuron, and therefore exhibit cognitive protecting effect.

Lipids are important essential macronutrients for energy storage and supply. Brain is the second largest lipid containing organ after adipose tissue, in which lipid-rich components are related to the disturbance of lipid metabolism homeostasis, and may participate in the occurrence and development of various neurodegenerative diseases (9). Besides, multiple interactions between abnormal lipid metabolism and AD pathogenesis have been found, including amyloidosis, oxidative stress, bioenergy deficits, neuroinflammation, and myelin degeneration (10). These results further highlight the significant association of lipid metabolism disorder and AD pathology.

To date, few studies have systematically explored the impact of high fat and/or VA nutritional status on cortical gene expression profile and Aβ metabolism. In the present study, APP/PS1 mice were fed with high fat diet containing different dosage of VA for 8 months. Transcriptomic sequencing was performed to in cerebral cortex to investigate the changes of gene expression profile, and the differential expressed genes (DEGs), especially the genes related Aβ metabolism, were further validated, and its role in modifying Aβ metabolism was explore *in vitro*. This study aimed to identify the potential functional molecules that contribute to the development of AD under the high fat and/or low VA nutritional status.

## Materials and methods

## Experimental animals and treatment

Three-week-old male APP/PS1 mice (*n* = 8 to 10 for each group) were obtained from experimental animal center of Capital Medical University (Beijing, China). Following acclimation, the mice were randomly divided into four intervention groups: (1) high fat, high sugar with low VA group (APP-FAT-LVA), (2) high fat, high sugar with normal VA group (APP-FAT-NVA), (3) normal fat, normal sugar with low VA group (APP-CON-LVA), (4) normal fat, normal sugar with normal VA group (APP-CON-NVA). The experimental diets were custom-formulated (SYSE BIO, China) based on the AIN-76A standard (Supplementary Table S1). The VA concentration was set at 4,000 IU/kg for the normal VA groups and 400 IU/kg for the low VA groups. The high fat, high sugar diet contained 45% fat (w/w) and 20% fructose (w/w). After 8 months of dietary intervention, with weekly monitoring of body weight and intake, mice were deeply anesthetized with 4% isoflurane and euthanized for cortical tissue collection. The animal experimental procedure was strictly accordance with the Guidelines for the Care and Use of Laboratory Animals, and was approved by the Animal Ethics Committee of Capital Medical University (AEEI-2020-012). The experimental timeline and workflow are illustrated in Figure 1A.

**Figure 1:**
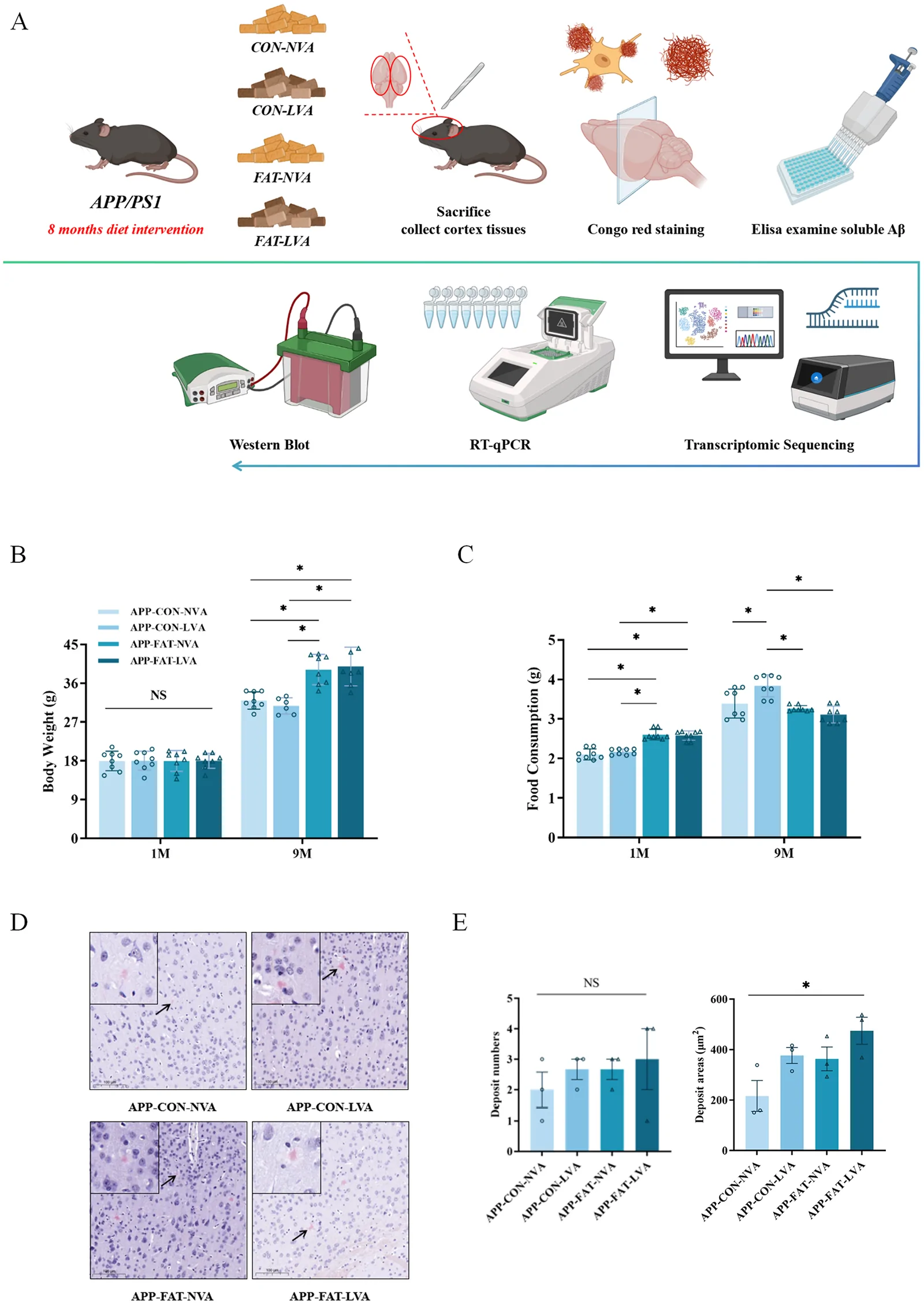
Impact of different diet and VA statuses on body weight, food intake, and cortical amyloid deposition in APP/PS1 mice. **(A)** Schematic diagram of the animal experimental design. **(B)** Body weight and **(C)** daily food intake of APP/PS1 mice under different dietary interventions. **(D)** Representative images of Congo red staining of amyloid deposits. The images are shown at a 100.0 μm field of view; scale bar = 50.0 μm (upper left). **(E)** Quantification of amyloid plaque number and areas. (*n* = 6–8 per group for all analysis, *n* = 3 mice per group for Congo red staining; 4 sagittal sections per mouse spaced 200 μm apart). Quantification analysis was performed using ImageJ in a blinded manner. APP, APP/PS1 mice; CON-NVA, normal fat, normal sugar with normal VA diet; CON-LVA, normal fat, normal sugar with low VA diet; FAT-NVA, high fat, high sugar with normal VA diet; FAT-LVA, high fat, high sugar with low VA diet. ^*^P < 0.05.

## Measurement of cortical Aβ plaque and Aβ content

The cortical Aβ plaque deposits in brain of APP/PS1 mice were detected by a Congo red staining (Key Gen Bio Tech, Wuhan, China) on paraffin-embedded sagittal sections (*n* = 3 animals per group). For each animal, 4 systematically sampled sagittal sections containing both the cerebral cortex and hippocampus (spanning lateral 1.0 mm to 2.0 mm relative to the midline, spaced 200 μm apart) were selected. Subregion boundaries for the cortex and hippocampus were strictly defined according to the standard Paxinos and Franklin mouse brain atlas. Brightfield digital images were acquired under constant exposure settings. Plaque load quantification was conducted using ImageJ software (NIH, Bethesda, MD, USA) by applying a uniform automated thresholding procedure across all samples to measure the percentage of plaque area relative to the total region of interest. All histological quantifications and image analyses were performed by an investigator blinded to the experimental group assignments. And the soluble Aβ_40_ and Aβ_42_ content in cortex were measured by Aβ ELISA high-sensitive kits (Wako, Tokyo, Japan).

## Transcriptomic sequencing

Total RNA was extracted from the mice cortex using TRIzol reagent (Sangon Biotech, Shanghai, China) following the manufacturer's instructions. RNA concentration and purity were assessed using the Qubit 2.0 (Invitrogen, Carlsbad, CA, USA) and Qubit 2.0 Fluorometer (Invitrogen, Carlsbad, CA, USA), ensuring an RNA Integrity Number (RIN) > 7.0 for all samples. All experiments were performed using independent biological replicates (*n* = 4–5 per group) processed and sequenced in a single batch to avoid technical bias. Poly(A) mRNA libraries were constructed using the Hieff NGS™ MaxUp Dual-mode mRNA Library Prep Kit for Illumina (YEASEN, Shanghai, China). Briefly, mRNA was purified via paramagnetic beads and thermally fragmented. First- and second-strand cDNA were synthesized, followed by end-repair, A-tailing, and adapter ligation. cDNA fragments of 300–500 bp were selected using Hieff NGS™ DNA Selection Beads. Sequencing was performed on the Illumina NovaSeq 6000 platform (Illumina, San Diego, CA, USA) with a paired-end 150 bp (PE150) strategy, generating at least 6 Gb of clean sequencing data per sample. Raw data quality was evaluated using Fast QC (https://www.bioinformatics.babraham.ac.uk/projects/fastqc/). Clean reads were aligned to the Mus musculus reference genome (GRCm38/mm10) using HISAT2 (v2.2.1). Gene expression levels were quantified as Fragments Per Kilobase of transcript per Million mapped reads using featureCounts (v2.0.1). Differential expression analysis and read count normalization (using the median-of-ratios method) were performed using the DESeq2 R package. To control the false discovery rate (FDR) across multiple pairwise comparisons, *p*-values were adjusted using the Benjamini-Hochberg procedure. Genes meeting the criteria of *q* value < 0.05 and |fold change (FC)| > 2 were identified as DEGs. To evaluate overall transcriptomic reproducibility and sample clustering, Principal Component Analysis (PCA) was performed on variance-stabilized transformation (VST) data using DESeq2.

## RNA extraction and quantitative real-time PCR

RNA was extracted with AIDpure Total RNA Kit (Aidlab, Beijing, China), and the quality of RNA was measured by microplate reader (Epoch, BioTek, USA). For cDNA synthesis, 1 μL RNA was reverse transcribed using the All-in-One RT Mixture IV (Aomei, Hangzhou, China). Real-time qPCR was performed on a CFX96 Real-Time PCR Detection System (Bio-Rad, Hercules, CA, USA) using SGExcel Fast SYBR Mixture (Sangon Biotech, Shanghai, China). Each 20 μL reaction consisted of 10 μL 2 × Taq Pro Universal SYBR qPCR Master Mix, 2 μL of cDNA template, 0.4 μL each of forward and reverse primers (10 μM), and 7.2 μL of ddH_2_O. The cycling parameters were as follows: 95 °C, 30 s; 40 cycles at 95 °C for 10 s, 60 °C for 30 s; and 65 °C for 5 s. Relative gene expression levels were normalized to *Gapdh* and calculated using the 2^−ΔΔ*Ct*^ method. Target gene functions and primer sequences are detailed in Supplementary Table S2 and Table 1, respectively.

**Table 1:** Primer sequences of RT-qPCR.

Genes	Primers	Sequence (5′-3′)
*ECE2*	*Forward* *Reverse*	AGCGGCGTGATGAGGAGAAG TACACCACCACAGGCTCAGAATC
*EIF4EBP2*	*Forward* *Reverse*	CCACCAGCCCAGCCAGAG CGGTCATAAATGATTCGTGTTCCTC
*FAM168B*	*Forward* *Reverse*	GGCTACACTCCTGGCACACC TTGCTGGGCGTATGGACTCTG
*GP1BB*	*Forward* *Reverse*	GCCTGCTGCTTCTGTTGCTAG GTGGTGTCGGGAGGGAAGG
*HES3*	*Forward* *Reverse*	ATCTCCAAGCCTCTGATGGAGAA AGCTTTCGTTTCCGTATCTGATG
*IFI27L2A*	*Forward* *Reverse*	TTGGGAACACTGTTTGGCTCTG GACATCATCTTGGCTGCTATGGAG
*IGIP*	*Forward* *Reverse*	GCAGTGTGTTGGGCTGTAATATAAC TTGGTTTCCACATGGTGAGTTCC
*PHIP*	*Forward* *Reverse*	GTTTACGCCTGTCTGCTTTCTTTG TCGCTTCTTCCTCTTCTTGCTTATG
*PRLR*	*Forward* *Reverse*	CACAGTAAATGCCACGAACGAAATG AGGCTCTGGTTCAACAATGTAAGTC
*SSTR4*	*Forward* *Reverse*	AGCGGGCATGGTCACTATCC GTCTTCATCTTGGCATAGCGTAGG
*TGTP1*	*Forward* *Reverse*	TGGGACCACTAACTTCACACC GGCCAGTTGTGCATCATTTTC
*UCN3*	*Forward* *Reverse*	AAGCCTCTCCCACAAGTTCTA GAGGTGCGTTTGGTTGTCATC

## Protein extraction and Western blot

Cerebral cortex tissues (20 mg per sample) were homogenized in RIPA lysis buffer (Beyotime, Shanghai, China) supplemented with 1 mM PMSF (Beyotime) and a phosphatase inhibitor cocktail (Biosharp, Hefei, China) at a 100:1:1 ratio. Protein concentrations were determined by using the BCA Protein Assay Kit (New cell and Molecular Biotech Co. Ltd, Suzhou, China). Equal amounts of protein (20 μg) were electrophoresed on Tris-tricine gradient gels and subsequently transferred onto PVDF membranes. The membranes were blocked and then incubated overnight at 4 °C with the following primary antibodies: ECE2 (1:250, Proteintech, Wuhan, China), HES3 (1:1,000, ImmunoWay, Plano, TX, USA), SSTR4 (1:1,000, ImmunoWay, Plano, TX, USA). After washing, the membrane was incubated with appropriate secondary anti-rabbit IgG (1:15,000, Cell Signaling Technology, Danvers, MA, USA) for 1 h at room temperature. Protein bands were visualized using an enhanced chemiluminescence system (Bio-Rad Laboratories, Hercules, CA, USA). Beta-Actin (1:1,000, Servicebio, Wuhan, China) or Beta-Tublin (1:1,000, Sino Biological, Beijng, China) served as the loading control. Densitometric analysis was performed using ImageJ software (version 1.54p; NIH, Bethesda, MD, USA).

## Cell culture and treatments

Human neuroblastoma SH-SY5Y cells (Procell, Wuhan, China) were maintained in Dulbecco's Modified Eagle's Medium (DMEM) supplemented with 10% fetal bovine serum (FBS) and 1% penicillin-streptomycin at 37 °C in a humidified 5% CO_2_ atmosphere. To induce neuronal-like differentiation, cells were pretreated with all-trans retinoic acid (ATRA; 10 μM, Meilunbio, China) for 72 h, following established neuronal differentiation protocols (11). Since RA is the primary bioactive metabolite of VA mediating physiological functions, it was finally utilized to treat cells instead of retinol *in vitro*, with concentrations of RA at 0.1 nM for the low-VA group and 10 nM for the normal-VA group. These concentrations were selected based on previous *in vitro* studies and were not intended to reproduce the systemic dietary conditions in mice.

## Aβ treatment

Differentiated cells were assigned to eight experimental groups to evaluate Aβ-induced toxicity. The four basal groups were established as follows: (1) control with low VA (CON-LVA); (2) control with normal VA (CON-NVA); (3) high-fat with low VA (FAT-LVA); and (4) high-fat with normal VA (FAT-NVA). High-fat conditions were induced by supplementing the medium with palmitic acid (PA; 200 μM, Meilunbio, Dalian, China) (12). The remaining four groups received additional treatment with 100 μM Aβ_25 − 35_ (TargetMol, Boston, MA, USA). Prior to use, the Aβ_25 − 35_ was dissolved in sterile water to prepare a 1 mM stock solution and incubated at 37 °C for 9 days to allow for pre-aggregation. Following a 48 h incubation under these respective conditions, ECE2 levels in both cells and culture media were quantified by ELISA.

## Effect of ECE2 activation on Aβ metabolism

The SH-SY5Y cells were divided into eight groups to assess the effects of ECE2 activation on Aβ generation. Cells from groups 1 to group 4 received the same treatments as the CON-LVA, CON-NVA, FAT-LVA, and FAT-NVA groups described above, respectively. Cells from groups 5 to group 8 were additionally treated with 0.1 μM of the ECE2 agonist Phosphoramidon disodium salt (CAS No. 119942-99-3, Catalog No. P141352, purity ≥ 97%; Shanghai Aladdin Biochemical Technology Co., Ltd., Shanghai, China). After 48 h of intervention, culture supernatants were collected for the measurement of ECE2, Aβ40, and Aβ4_2_ via ELISA. Total cellular proteins were harvested to determine the expression of Aβ metabolism related proteins, including APP, IDE, ADAM10, and BACE1, via Western blot using specific antibodies (Abcam, Cambridge, UK).

## Statistical analysis

Data was expressed as the mean ± SD. Statistical analysis was performed by SPSS 26.0 (SPSS, Inc., Chicago, IL, USA) and the images were made by Graph Pad Prism 8 (GraphPad Software Inc., San Diego, CA, USA). Data were analyzed using one-way ANOVA and Tukey's Honest Significant Difference Test for post comparison. For the animal experiments with a 2 × 2 factorial design, two-way ANOVA was performed to evaluate the main effects of dietary condition (FAT), VA status, and their interaction (FAT × VA) on gene and protein expression. *P-*value < 0.05 was considered as statistically significant.

## Results

### Animal experiments

#### Body weight and daily dietary intake of mice treated with different diets

During 8 months dietary intervention, mice fed with high fat and high sugar diet displayed significantly higher body weights compared with the mice fed with control diet (Figure 1B), whereas the content of VA in diets seems has no effect on weight gain in mice. At 9 months of age, daily food intake was significantly higher in the APP-CON-LVA group than in the APP-CON-NVA group. Mice from both APP-FAT-LVA and APP-FAT-NVA groups exhibited significantly lower daily feed consumption as comparing with the APP-CON-LVA group (Figure 1C).

## Cortical Aβ plaque and Aβ content in mice treated with different diets

We did not find significant inter-groups differences in the number of cortical Aβ plaque (Figures 1D, E). While plaque areas in the cortex of mice from APP-CON-LVA and APP-FAT-NVA groups displayed an increased trend as comparing with the APP-CON-NVA group, and a significant increase was observed exclusively in the mice from APP-FAT-LVA group (*P* < 0.05). Soluble Aβ_42_ content in cortex remained stable across all groups, whereas in mice fed with low VA containing diet showed significantly reduced cortical Aβ_40_ level and elevated Aβ_42_/ Aβ_40_ ratio (Table 2).

**Table 2:** Soluble Aβ in the cortex of the 9 months APP/PS1 mice.

Parameters	APP-CON-NVA	APP-CON-LVA	APP-FAT-NVA	APP-FAT-LVA	*p*-value
Aβ_1 − 40_ (ng/mg)	32.60 ± 4.83	19.67 ± 3.07 ^a^	28.08 ± 2.00	15.65 ± 1.11 ^ac^	0.001
Aβ_1 − 42_ (ng/mg)	31.79 ± 2.56	34.09 ± 1.81	35.17 ± 1.88	36.02 ± 3.21	0.245
Aβ_1 − 42_/Aβ_1 − 40_	0.99 ± 0.15	1.77 ± 0.36 ^a^	1.26 ± 0.10	2.30 ± 0.07 ^ac^	<0.001

One-Way ANOVA was used for group comparison and Bonferroni test was used for post comparison. *n* = 3 for each group. a: compared with APP-CON-NVA group; b: compared with APP-CON-LVA group; c: compared with APP-FAT-NVA group.

## Identification of differentially expressed genes

Following stringent quality control and genomic alignment (Supplementary Tables S3, S4), transcriptomic profiling identified differentially expressed genes based on criteria of *q* Value < 0.05 and | FC | > 2 (Figure 2, Supplementary Table S5). To evaluate sample reproducibility and overall transcriptomic relationships across groups, Principal Component Analysis (PCA) was performed on variance-stabilized read counts (Supplementary Figure S1). Biological replicates within each group clustered tightly together, showing clear separation between treatment conditions without noticeable outliers, confirming the high quality and consistency of the sequencing data. Among 40,175 enriched genes, the comparison between APP-FAT-LVA and APP-CON-LVA groups yielded 110 DEGs, comprising 63 up-regulated and 47 down-regulated genes. We found that *Gp1bb* and *Ifi27l2a* were significantly up-regulated, while *Hes3, Phip* and *Sstr4* gene expression were significantly down-regulated. Other comparisons revealed fewer transcriptomic alterations, such as the 19 DEGs (7 up-regulated, 12 down-regulated) observed between APP-FAT-LVA and APP-CON-NVA, where *Ifi27l2a* and *Tgtp1* expression increased, whereas *Eif4ebp2* and *Phip* expression decreased. Additionally, APP-FAT-NVA showed 11 DEGs relative to the APP-CON-NVA group, characterized by elevated *Igip* expression and candidate transcript alterations in *Ece2, Gfod2*, and *Sstr4*. Minimal DEGs were identified between APP-CON-NVA and APP-CON-LVA groups, as well as between APP-FAT-NVA and APP-CON-NVA cohorts, with notable variations in *Ece2, Prlr, Ucn3*, and *Fam168b* gene expression. To verify these candidate transcriptomic changes, targeted RT-qPCR and Western blot analyses were performed in subsequent sections.

**Figure 2:**
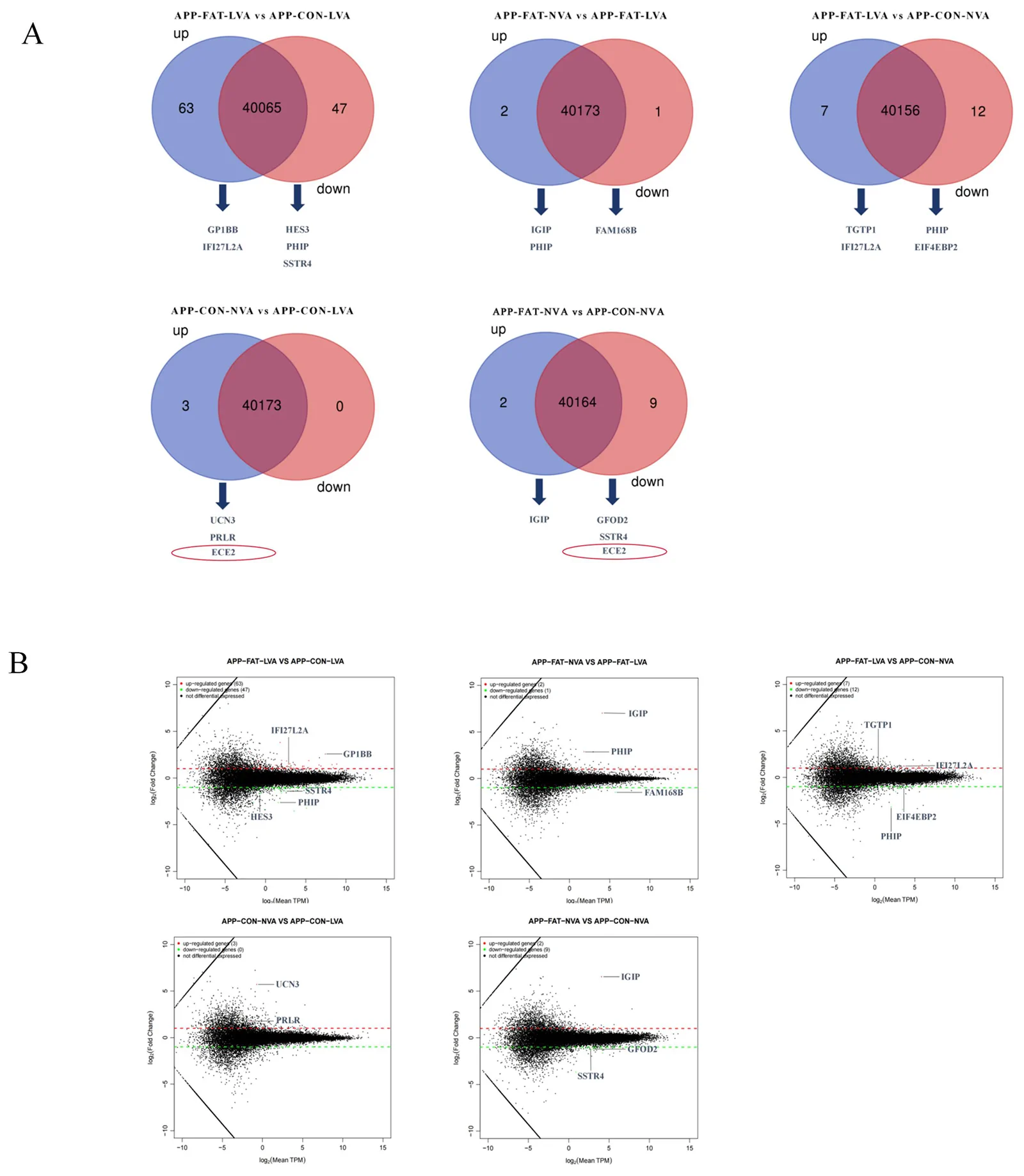
Transcriptomic analysis of differentially expressed genes in the cortex of APP/PS1 mice under different diet and VA statuses. **(A)** Venn diagram and **(B)** M-vs.-A plots showing the DEGs in each comparison (*n* = 4–5 per group). Blue part, up-regulated genes and their number; Red part, down-regulated genes and their number; The overlap of the blue and red part, the genes and their numbers that are not significantly changed in each group. Ece2 is not displayed within the plotting range because of its exceptionally large absolute log2 fold-change value. Mean TPM, grouping expression; log2 (Mean TPM), the mean log2 (TPM) of the two groups of samples, i.e., [log2 **(A)** + log2 **(B)**] /2; log2 (Foldchange), indicates the log2 (B/A) value, where A is the former group and B is the latter group. APP, APP/PS1 mice; CON-NVA, normal fat, normal sugar with normal VA diet; CON-LVA, normal fat, normal sugar with low VA diet; FAT-NVA, high fat, high sugar with normal VA diet; FAT-LVA, high fat, high sugar with low VA diet.

## Validation of DEGs

To cross-validate the transcriptomic findings, real-time qPCR was employed to determine the mRNA levels of the selected DEGs in the cortex of APP/PS1 mice (Figure 3A). Consistent with the sequencing data, high fat, high sugar treatment significantly up-regulated the expression of *Ece2, Sstr4, Hes3* and *Prlr*. Similarly, low VA containing diet treatment promoted the gene expression of *Hes3, Prlr*, and *Phip*, while down-regulating *Fam168b* expression. Notably, a synergistic interaction of HFD and low VA treatment on *Hes3* and *Prlr* gene expression was identified in cortex of APP/PS1 mice. Furthermore, the regulatory effects of diets containing different level of VA on cortical *Eif4ebp2, Tgtp1*, and *Ucn3* gene expression were modulated by dietary fat content, reflecting a potential interplay between these nutrients. Two-way ANOVA revealed significant effects of dietary fat and sugar, VA status, and their interaction on the expression of several candidate genes (Figure 3B). Notably, *Ece2* expression was significantly affected by dietary fat (*P* < 0.001) and VA status (*P* = 0.021), with a significant FAT × VA interaction (*P* = 0.001). Significant FAT × VA interactions were also observed for *Ifi27l2a, Phip, Prlr, Eif4ebp2, Tgtp1*, and *Ucn3* (*P* < 0.05).

**Figure 3:**
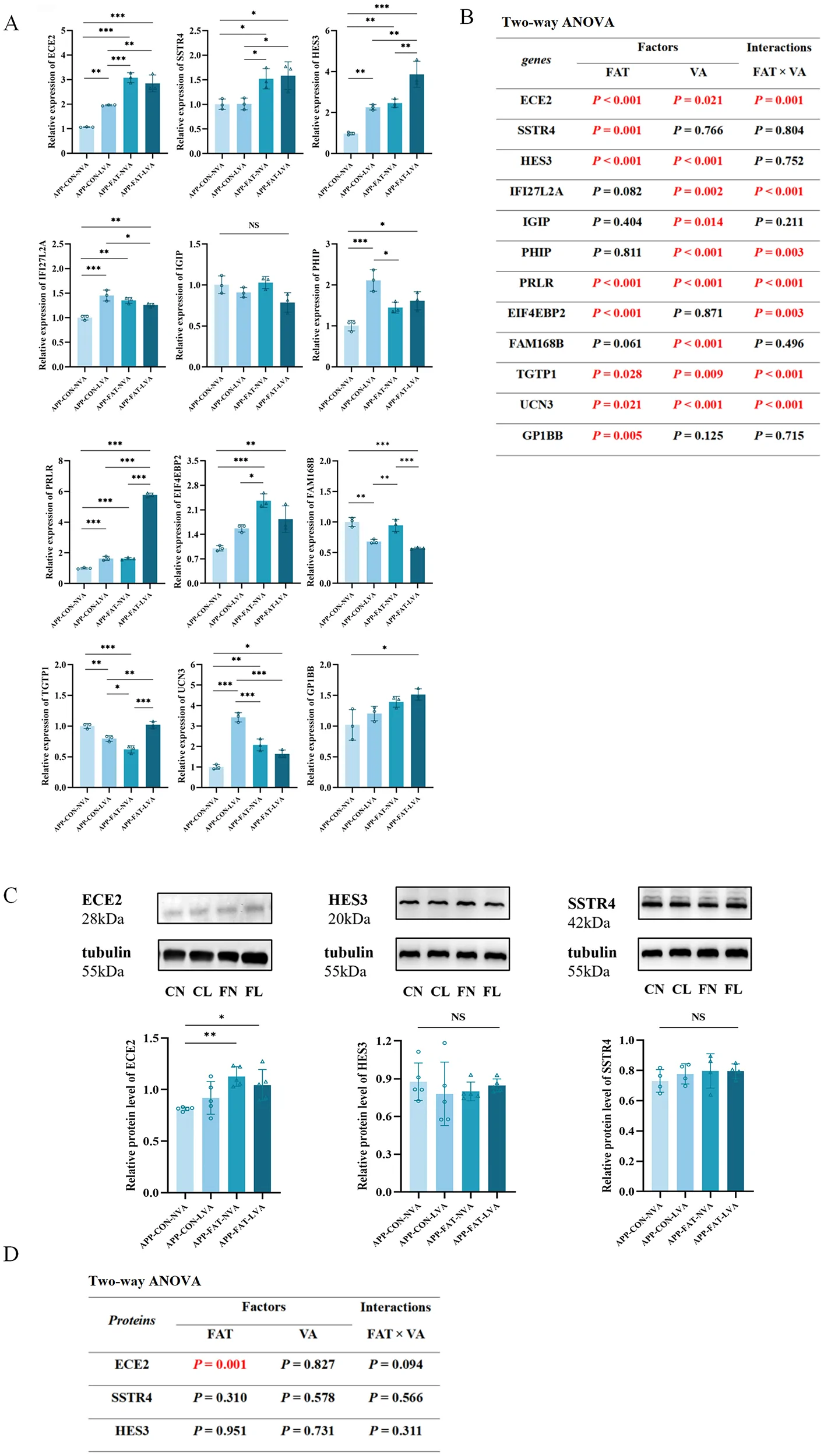
Targeted validation of selected candidate genes in the cortex of APP/PS1 mice. **(A)** RT-qPCR analysis of mRNA expression levels of selected candidate genes and **(B)** corresponding two-way ANOVA main effects and interaction analysis. **(C)** Western blot validation of selected proteins and **(D)** quantitative analysis with two-way ANOVA evaluation. Data were presented as mean ± SD. For multiple group comparisons, one-way ANOVA and Tukey's Honest Significant Difference Test were used in the comparison of groups. Two-way ANOVA was used to the main effects of diet (FAT) and VA status, as well as their interactive effect (FAT × VA). *n* = 3 for RT-qPCR and *n* = 4–5 for WB analysis. APP, APP/PS1 mice; CON-NVA, normal fat, normal sugar with normal VA diet; CON-LVA, normal fat, normal sugar with low VA diet; FAT-NVA, high fat, high sugar with normal VA diet; FAT-LVA, high fat, high sugar with low VA diet. *P < 0.05, **P < 0.01, ***P < 0.001.

## Protein expression of DEGs

Western blotting was performed to quantify the protein expression of the selected DEGs (Figure 3C). Among these candidate targets, only ECE2 exhibited significant protein-level alterations in response to dietary intervention. As a zinc-dependent metallopeptidase primarily expressed in neurons, ECE2 plays an important role in Aβ peptide degradation and neuropeptide processing (13, 14). Earlier evidence indicated that impaired ECE2 enzymatic function accelerated cerebral Aβ accumulation and worsened AD pathology, whereas its activation promoted Aβ clearance and reduced amyloid burden (15). The importance of ECE2 in maintaining amyloid homeostasis is further underscored by mutations in its peptidase domain which drastically impair catalytic efficiency (16). Notably, no significant interaction between dietary fat and VA content on cortical ECE2 protein expression was detected (Figure 3D). Furthermore, low VA containing diet treatment did not induce discernible changes in the cortical ECE2, HES3, or SSTR4 expression across the groups.

## *In vitro* experiment

## Modulation of ECE2 expression by Aβ_25 − 35_ and an ECE2 agonist

The expression levels of ECE2 in SH-SY5Y cells exhibited significant variability across the groups (Figure 4B). Comparing with the control groups, cells treated with high fat showed significantly higher ECE2 levels, whereas VA nutritional status had no significant impact on ECE2 expression. Upon Aβ_25 − 35_ treatment, ECE2 levels were markedly up-regulated in the CON-LVA, FAT-LVA, and FAT-NVA groups. Furthermore, treatment with an ECE2 agonist significantly elevated ECE2 protein levels across groups (*P* < 0.01).

**Figure 4:**
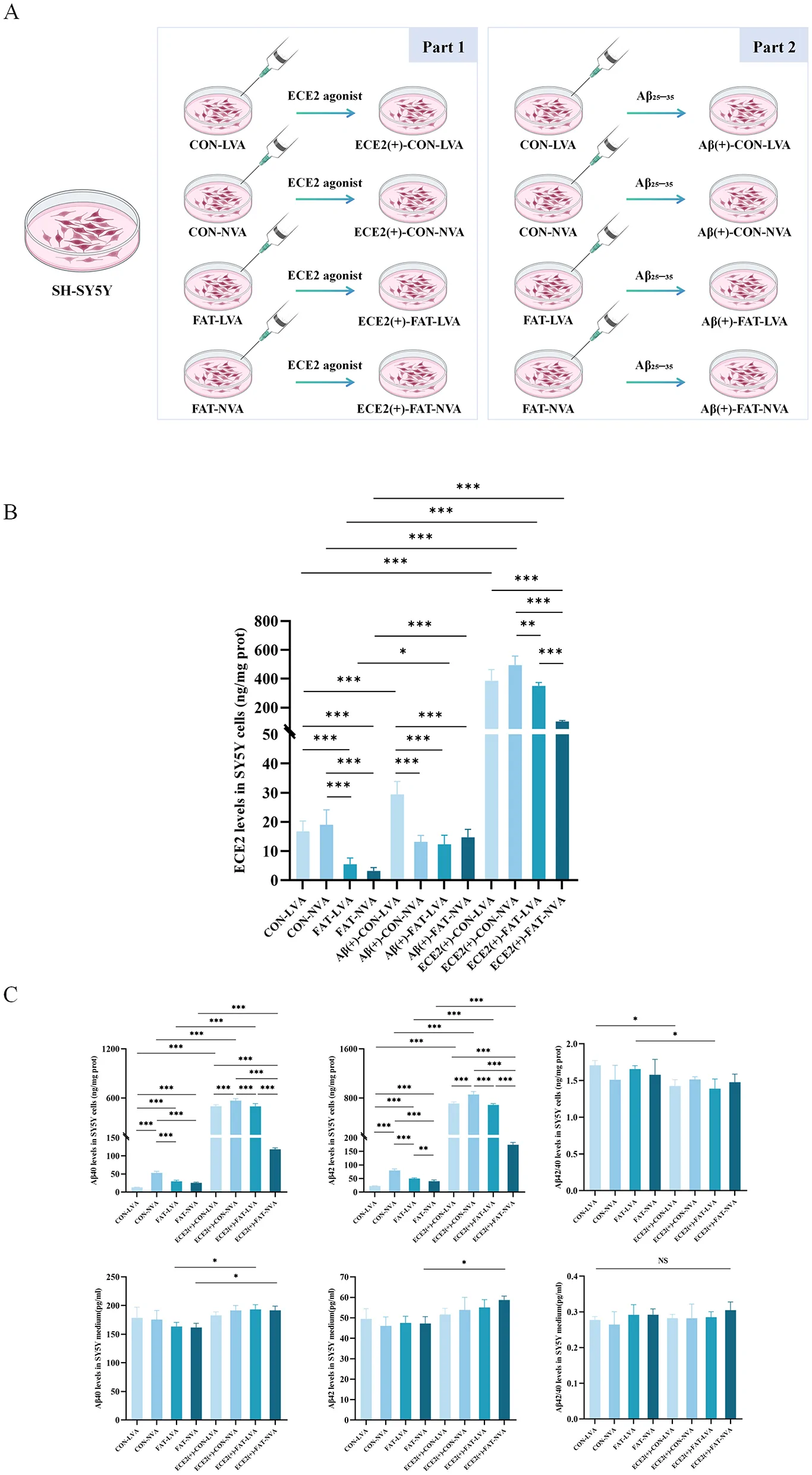
Effects of ECE2 modulation on Aβ metabolism in SH-SY5Y cells under different nutritional conditions. **(A)** Schematic overview of the *in vitro* experimental design. **(B)** Changes in ECE2 expression in response to Aβ stimulation and ECE2 agonist treatment under different nutritional conditions. **(C)** Intracellular and extracellular Aβ levels of SH-SY5Y cells after ECE2 agonist treatment. Data are presented as mean ± SD, (*n* = 3 independent experiments). One-way ANOVA and Tukey's Honest Significant Difference Test were uesd in the comparison of groups. Aβ, amyloid-beta; VA, vitamin A; CON-NVA, normal fat + normal VA medium; CON-LVA, normal fat + low VA medium; FAT-NVA, high fat + normal VA medium; FAT-LVA, high fat + low VA medium. * *P* < 0.05, ** *P* < 0.01, *** *P* < 0.001.

## Impact of ECE2 activation on Aβ metabolism

As shown in Figure 4C, the lowest and the highest baseline Aβ_40_ and Aβ_42_ levels in SH-SY5Y cell were found in the CON-LVA and CON-NVA group respectively. Following the treatment of ECE2 agonist, intracellular Aβ_40_ and Aβ_42_ levels significantly increased across all groups (*P* < 0.001). After ECE2 activation, the cell received high fat treatment exhibited significantly lower Aβ_40_ and Aβ_42_ concentrations as comparing with the other three groups. While the intracellular Aβ_42_/Aβ_40_ ratio remained largely consistent across groups, ECE2 activation selectively reduced this ratio in the cells from CON-LVA and FAT-LVA groups.

In the culture medium, ECE2 activation was associated with increased extracellular Aβ_40_ levels in both FAT-LVA and FAT-NVA groups, with a concomitant increase in Aβ_42_ specifically observed in the FAT-NVA group. However, the extracellular Aβ_42_/Aβ_40_ ratio showed no significant inter-group differences regardless of different treatments (*P* > 0.05).

## Effect of ECE2 activation on Aβ metabolism-related genes expression

Following treatment with the ECE2 agonist, *APP* mRNA expression was significantly down-regulated in the cells from CON-NVA, FAT-LVA, and FAT-NVA groups (Figure 5A). ECE2 activation induced divergent responses in *IDE* gene expression, characterized by a significant up-regulation in the CON-NVA group, but marked suppression in both FAT-NVA and FAT-LVA groups. Furthermore, *ADAM10* mRNA expression was significantly attenuated in the FAT-NVA group upon ECE2 agonism. Similarly, a significant reduction in *BACE1* expression was observed in the cells from FAT-LVA and FAT-NVA groups following agonist treatment.

**Figure 5:**
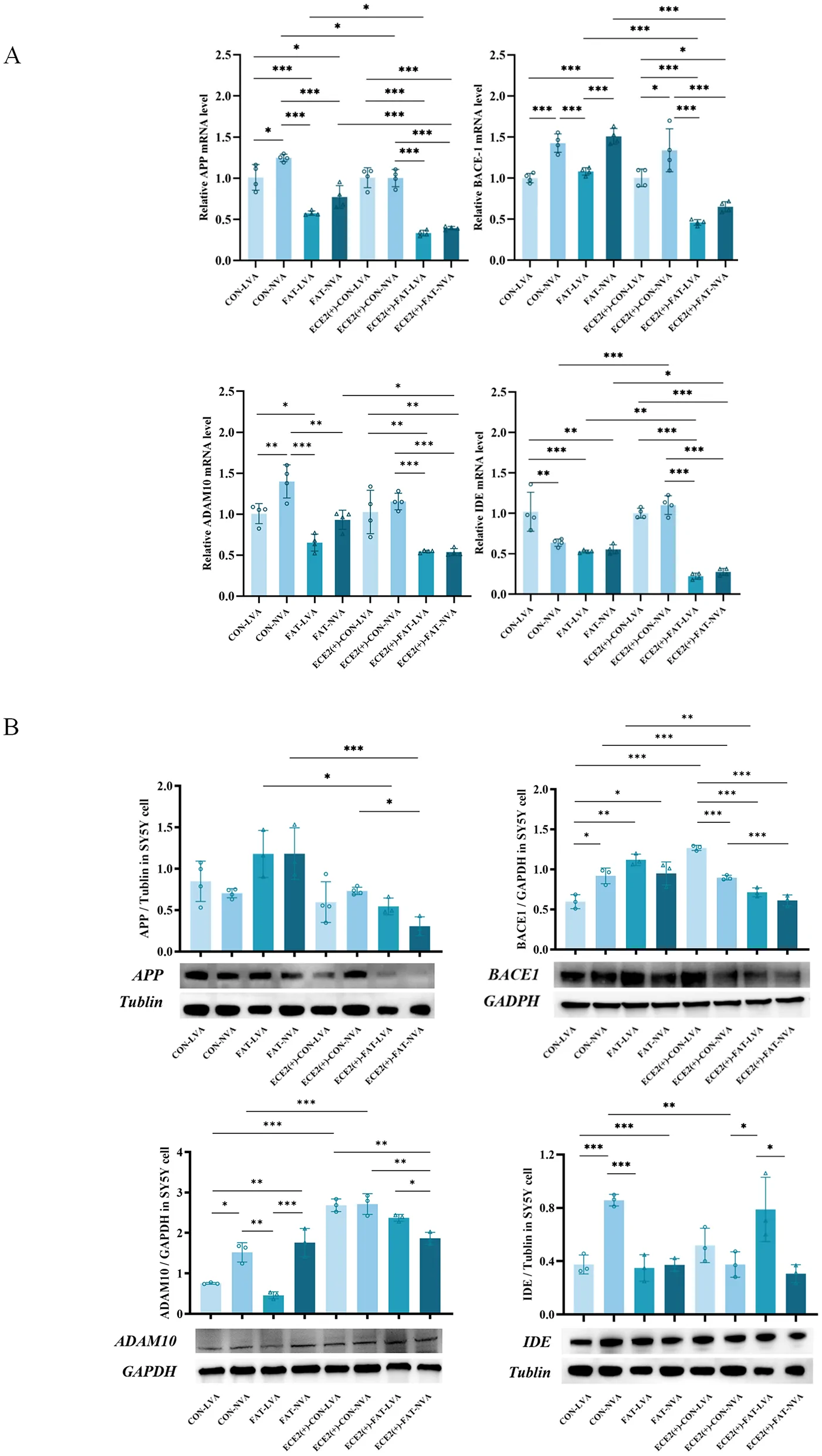
Effects of ECE2 agonist treatment on Aβ metabolism-related gene and protein expression in SH-SY5Y cells. **(A)** Relative mRNA expression of Aβ metabolism-related genes in SH-SY5Y cells after ECE2 agonist treatment under different nutritional conditions. **(B)** Western blot of Aβ metabolism-related proteins after ECE2 agonist treatment. Data were presented as mean ± SD. One-way ANOVA and Tukey's Honest Significant Difference Test were used in the comparison of groups. *n* = 4 per group for RT-qPCR and *n* = 3–4 per group for Western blot analysis. Aβ, amyloid-beta; VA, vitamin A; CON-NVA, normal fat + normal VA medium; CON-LVA, normal fat + low VA medium; FAT-NVA, high fat + normal VA medium; FAT-LVA, high fat + low VA medium. * *P* < 0.05, ** *P* < 0.01, *** *P* < 0.001.

## Effect of ECE2 activation on Aβ metabolism-related protein expression

ECE2 agonism significantly down-regulated APP protein expression in FAT-LVA and FAT-NVA groups (Figure 5B). IDE protein expression showed divergent trends, with significant attenuation in CON-NVA group, but up-regulation in FAT-LVA group. Similarly, increased BACE1 protein expression was found in CON-LVA group, while significantly decreased expression was observed in CON-NVA and FAT-LVA groups. Conversely, ADAM10 protein expression was consistently up-regulated across CON-LVA, CON-NVA, and FAT-LVA groups.

## Discussion

We performed transcriptomic profiling to map the cortical landscape of APP/PS1 mice under high fat and low VA nutritional status. We detected a total of 40,175 genes and identified a distinct set of DEGs. These candidate DEGs, particularly those associated with neuronal health and amyloid metabolism, were subsequently validated through RT-qPCR and Western blot. High-throughput RNA sequencing provided valuable candidate gene targets across different dietary interventions. While initial transcriptomic pipelines can occasionally display localized variations due to sequencing depth or background noise, our comprehensive multi-level validations, including RT-qPCR, Western blotting, and *in vitro* cellular assays, consistently established that high-fat diet intervention significantly up-regulates ECE2 expression at both transcript and protein levels. Overall, our data demonstrate that dietary fat and VA nutritional status remodel the cortical transcriptome in this AD model. Among these changes, the regulation of the amyloid-related *Ece2* gene highlights a specific molecular response to the interplay between dietary fat intake and VA nutritional status.

*Ece2* emerged from our analysis as a gene notably sensitive to both high-fat intake and VA status. Specifically, HFD triggered a significant upregulation of *Ece2* mRNA within the cortex of APP/PS1 mice. Study has found that ECE2 facilitates the production of various bioactive peptides including bovine adrenal medulla peptides 22 and 12 (17). These molecules might accumulate in GABAergic neurons, where they modulate neuroendocrine distribution and intracellular peptide biosynthesis (18). Results from the study of *Ece2* knockout models have revealed significant deficits in spatial learning and memory, which may be caused by abnormal extracellular matrix secretion and protein phosphorylation (19). Since high-fat and VA deficient diets are possible to accelerate Aβ generation and increase AD susceptibility (20, 21), the induction of *Ece2* mRNA expression likely indicated a possible compensatory response associated with Aβ stress, although its functional consequence remains uncertain. This biological relevance is reinforced by the elevated cortical ECE2 protein expression observed in HFD-treated APP/PS1 mice.

Our study also identified several DEGs integral to neurodevelopment and myelin homeostasis, most notably the *Hes3* and *Fam168b* genes. HES3 is a basic helix-loop-helix transcriptional repressor that regulates neural stem cell (NSC) maintenance, the timing of neurogenesis, and overall brain regenerative capacity (22). Findings elsewhere indicate that HES3 deficiency is implicated in premature neuronal differentiation and reduced expression of myelin-associated proteins (23). Furthermore, HES3 associated with NSC renewal and brain regeneration by promoting insulin release during the developmental phase (24). In the current study, we found that cortical *Hes3* mRNA expression increased significantly following high-fat and low VA dietary intervention with a synergistic enhancement observed specifically in the FAT-LVA group. However, we failed to find the corresponding alterations in HES3 protein expression, implying that the dietary regulative effect of different diets on HES3 expression is largely on the transcriptional level.

As a major regulator of myelin integrity and axonal function, *Fam168b* is predominantly localized to the central nervous system, and its down-regulation is linked to myelin defects and impaired neuronal signaling (25). *Fam168b*, also known as myelin-associated neurite-outgrowth inhibitor (MANI), has been shown to regulate neuronal neurite and axonal growth (26, 27). In the present study, we found that low VA containing diet significantly inhibited cortical *Fam168b* mRNA expression, while HFD exerted no discernible effect, suggesting that *Fam168b* was a sensitive gene to VA nutritional status may be associated with neuronal and myelin homeostasis. These findings indicated that chronic VA deficiency-medicated inhibition of *Fam168b* expression might involve in the pathological progression of AD through triggering neuronal injury and inducing myelin defects.

Multiple differentially expressed genes within our dataset including *Prlr, Ucn3*, and *Sstr4* serve as pivotal components of metabolic and neuroendocrine signaling networks. PRLR belongs to the class I cytokine receptor superfamily and facilitates prolactin signaling. This receptor is known to influence the development of insulin resistance through regulating the JAK/STAT pathway (28). UCN3 is a peptide hormone within the corticotropin-releasing factor family that functions as a regulator of insulin secretion and energy balance (29). In our study, the significant elevation of cortical *Prlr* and *Ucn3* mRNA expression following low VA dietary intervention suggests a possible association between VA nutritional status and insulin-related signaling. These molecular shifts should be regarded as candidate to the metabolic dysregulation observed during the progression of AD pathology.

SSTR4 is predominantly localized to the cortex and hippocampus, where it has been implicated in neuroendocrine signaling and neuroinflammatory responses (30). Investigations in *Sstr4* deficient mice have demonstrated the up-regulation of the Calcitonin Gene-Related Peptide (*Cgrp*) gene (31). This peptide functions as a potent mediator of neurogenic inflammation by triggering the release of neuronal sensitizers from mast cells (32). These associations emphasize the importance of SSTR4 in regulating calcium homeostasis and neuroinflammation. Furthermore, the pharmacological blockade of SSTR4 has been shown to attenuate hyperlipidemia induced neuronal damage in APP/PS1 models (33). Our results indicate that HFD fed APP/PS1 mice exhibited significantly elevated cortical *Sstr4* expression, but not affected by low VA diet intake, suggesting that *Sstr4* may be preferentially responsive to lipid metabolic stress rather than VA deficiencies.

By integrating omics screening with experimental validation in the APP/PS1 mice cortex, we identified ECE2 as a potential regulatory candidate. We further explored its functional and mechanistic roles in SH-SY5Y cells through pharmacological activation of ECE2. The results demonstrate a clear association between ECE2 activity and Aβ dynamics. Crucially, the impact of ECE2 on Aβ homeostasis appears to be context dependent and is heavily governed by the underlying nutritional environment. Aβ treatment markedly induced ECE2 expression in SH-SY5Y cells, demonstrating the likely participation of ECE2 in cellular stress responses or compensatory pathways triggered by amyloid accumulation. The concurrent modulation of ADAM10 and BACE1 expression suggests that ECE2′s regulatory influence spans the secretory and endocytic pathways. These findings converge with evidence that ECE family members regulate intracellular Aβ pools within endosomal and lysosomal compartments, where the acidic microenvironment supports both the proteolytic activity of ECE enzymes and the processing of amyloid precursors (34). Earlier research has documented that Aβ exposure-mediated *Ece2* transcription in SH-SY5Y cells follows a specific temporal curve with an initial decline followed by a subsequently rebound (35). This expression trajectory suggests that the eventual increase of *Ece2* expression hints a delayed compensatory mechanism to mitigate Aβ load in brain, further reinforce the correlation between cerebral amyloid dynamics and ECE2 (36).

Contrary to expectations, ECE2 activation did not reduce Aβ production, but led to an overall increase in SH-SY5Y cells. While both intracellular Aβ_40_ and Aβ_42_ concentrations increased following ECE2 activation with a decline of Aβ_42/40_ ratio. These results indicate that the impact of ECE2 on Aβ homeostasis involves more than simple quantitative shifts and includes a relative redistribution of Aβ species toward Aβ_40_ (37). These alterations likely derived from a perturbation of the regulatory networks that maintain Aβ metabolism balance. Except of enhancing clearance capacity, ECE2 activation appears to induce a reorganization of the processes governing Aβ generation, trafficking, and degradation (38). This remodeling may alter the equilibrium of Aβ distribution between intracellular and extracellular compartments. Therefore, ECE2 activation might facilitate the delivery of Aβ into the endocytic lysosomal processing pathway. If the flux through this pathway is limited or if clearance mechanisms reach saturation, net Aβ removal may not increase in proportion to ECE2 activity. Under these circumstance, the prolonged activation of ECE2 could eventually result in the accumulation of intracellular Aβ (39).

Consistent with these results, ECE2 activation caused significant changes of key enzymes involved in Aβ metabolism in the transcriptional and translational levels. We observed a decline in APP and BACE1 expression, but an increase in ADAM10 protein expression, demonstrating the role of ECE2 activation in remodeling Aβ metabolism toward the non-amyloidogenic processing pathway by regulating APP cleavage dynamics (40).

It was reported that BACE1 expression and activity are governed by multiple signaling pathways and transcription factors. Furthermore, its protein abundance is influenced by posttranslational modifications and subcellular localization. We therefore speculated that the discrepancies between BACE1 and ADAM10 at the mRNA and protein levels might be attributed to the mechanisms beyond transcriptional regulation including posttranscriptional control and protein stability (41). Besides, ADAM10 activity is more heavily influenced by intracellular trafficking and proteolytic processing (42). These distinct regulatory frameworks likely lead to the divergent responses observed following ECE2 activation. In our study, despite the absence of parallel reductions in Aβ levels relative to changes in production, ECE2 is associated with Aβ dynamics and may contribute to the regulation of Aβ homeostasis, but the present study does not establish a causal role. Previous studies have shown that ECE2 expression is significantly increased in the brains of AD patients, and is closely linked to Aβ degradation, supporting its role in compensatory clearance of Aβ (35). Additionally, study also fund that loss of ECE2 function reduces the ability to degrade Aβ, in contrast, increase in ECE2 levels can lower amyloid burden and improve pathological outcomes (16).

The Aβ degradation capacity of brain serves as a critical determinant of Aβ homeostasis. Our results show that activation of ECE2 coincides with a reduction in IDE protein levels. These findings suggest that the engagement of ECE2 within the Aβ clearance network may trigger a redistribution or feedback regulation among various degrading enzymes. While ECE2 is not typically viewed as the primary Aβ degrading enzyme, it functions within an interconnected clearance system alongside partners such as IDE (43). Importantly, ECE2 and IDE operate in distinct cellular environments. ECE2 primarily functions within acidic endocytic and lysosomal compartments, while IDE exhibits higher activity in neutral cytoplasmic or extracellular settings (34). This spatial partitioning creates a scenario for substrate competition. Upon ECE2 activation, Aβ substrates may be preferentially directed toward ECE2 mediated pathways and thus indirectly reduce the substrate availability for IDE. These results align with previous reports of altered enzyme expression and activity in brain of AD patients (44). Furthermore, differences in cellular localization and substrate allocation are known to influence overall clearance efficiency of Aβ (45). Consequently, the activation of ECE2 may reduce the functional reliance on IDE mediated degradation by rerouting Aβ substrates and eliciting compensatory adjustments across the clearance network.

Crucially, our observations demonstrate that high fat and VA nutritional status function as fundamental modifiers of the ECE2 associated Aβ metabolism regulatory axis. Variations in nutritional status likely alter metabolic stress and protein homeostasis which in turn impacts the capacity of cellular degradation and transport systems of Aβ (46). This interplay determines the specific magnitude and direction of ECE2 on Aβ stability. Under high fat condition, ECE2 activation did not achieve a reduction in Aβ, and instead led to a concomitant rise in both intracellular and extracellular Aβ pools. These results suggest that lipid metabolic disturbance might compromise the degradative function of ECE2 through the broader dysfunction of the endosomal lysosomal system (47). Saturated fatty acids has been proposed to inhibit clathrin-mediated endocytosis, and thus limit the trafficking of Aβ and its precursors to primary degradation sites (48). Moreover, impaired lysosomal acidification combined with compromised membrane integrity have been reported to reduce the total proteolytic capacity of the cells (49). Under this environments, even the targeted activation of ECE2 may not be sufficient to enhance Aβ clearance due to disrupted substrate delivery or the loss of the acidic microenvironment necessary for optimal enzymatic activity. This hypothesis requires direct experimental validation.

On condition of VA deficiency, ECE2 activation resulted in elevated total Aβ levels accompanying with a reduced Aβ_42/40_ ratio. These results are consistent with the possibility that disruption of retinoic acid signaling which may be associated with oxidative stress and enhances BACE1 expression (50). Under this specific VA nutritional environment, we speculate that ECE2 activation might favor the clearance of hydrophobic Aβ_42_ or its conversion into the less toxic Aβ_40_ isoform (51). Such selective dynamics explain the decrease in the Aβ_42/40_ ratio. Nevertheless, this putative effect may not be strong enough to offset the increase in Aβ generation associated with elevated BACE1 levels. Although, the parallel up-regulation of ADAM10 indicated an effort to re-balance APP processing, it cannot overcome the primary amyloidogenic initiated by BACE1 over-expression. This disturbance of APP cleavage balance eventually leads to a net accumulation of Aβ.

Under combined high fat and VA deficient conditions, ECE2 activation increased intracellular levels of Aβ_40_ and Aβ_42_ with reduced Aβ_42/40_ ratio. However, only extracellular Aβ_40_ level showed a significant elevation, suggesting that lipid metabolic disturbance and VA deficient interact to reconfigure the production and distribution of soluble Aβ (52). Although the coordinated down-regulation of APP, BACE1 and the up-regulation of ADAM10 reflect a cellular effort to favor non-amyloidogenic processing, the total Aβ production remained elevated.

It has been proposed that high fat induced remodeling of lipid rafts can enhance BACE1 enzymatic activity (53) and simultaneously modulate γ secretase function to favor Aβ_40_ generation over Aβ_42_ (54). Simultaneously, impaired retinoic acid signaling and oxidative stress caused by VA deficiency have been suggested to further drive BACE1 activity through JNK signaling pathways (55). These conditions also promote posttranslational modifications of Aβ_42_ that accelerate its aggregation from soluble monomers (56). This process likely reduces the detectable intracellular Aβ_42_ and partially explains the observed decrease in the Aβ_42/40_ ratio.

IDE expression showed no significant change under high fat and VA deficient status, indicating that extracellular degradation pathways failed to activate effectively (57). The simultaneous impairment of endocytic lysosomal function triggered by high fat conditions (58) and the oxidative damage linked to VA deficiency (59) likely act in synergy to restrict the intracellular degradative capacity of ECE2. When ECE2 undergoes activation, its functional efficacy remains constrained by hindered substrate delivery and disrupted lysosomal acidification alongside potential substrate competition with IDE (43). Additionally, dysfunction within the endosome Golgi recycling pathway has been hypothesized selectively obstruct Aβ_42_ efflux (60), while Aβ_40_ may escape through less affected routes such as passive diffusion (61). These mechanisms provide a plausible explanation for the selective increase in extracellular Aβ_40_ recorded under these specific nutritional conditions.

The innovation of this study lies in its integration of a long-term dietary intervention conducted in AD model mice with transcriptomic analysis, which systematically elucidates the collective impact of VA nutritional status and high fat intake on cortical gene expression. By merging omics-based screening with targeted functional validation, we identifies ECE2 as a pivotal regulator of Aβ metabolism in response to high fat and VA deficiency in the pathology of AD.

Several limitations of this study should be acknowledged. The transcriptomic profiling was restricted to the cerebral cortex because the available hippocampal samples were insufficient for parallel sequencing. This limitation precluded a comparative analysis between distinct brain regions. We acknowledge that the modest sample size (4–5 samples per group) presents potential statistical power limitations for low-abundance transcripts. Thus, our global RNA-seq profiling and bioinformatic network analyses should be interpreted as exploratory and hypothesis-generating. Crucially, key mechanistic targets identified from this screen were orthogonally validated using Western Blot, establishing robust confirmatory evidence. Histological evaluation of amyloid pathology in this study relied on Congo red staining and biochemical ELISA measurement of soluble Aβ_40/42_. While Congo red effectively highlights dense-core fibrillar amyloid plaques within the cortex and hippocampus, it is relatively less sensitive for detecting early diffuse amyloid deposits compared to Aβ immunohistochemistry (such as 6E10 or Thioflavin-S). Future investigations incorporating 6E10 immunostaining will be valuable for further characterizing early-stage diffuse plaque dynamics under diverse dietary interventions. Furthermore, although ECE2 expression was evaluated at the mRNA and protein levels, and its potential role in Aβ metabolism was further investigated *in vitro*, we did not directly measure the enzymatic activity of ECE2 or the specific bioactive peptide substrates and products *in vivo*. Therefore, the present findings cannot establish whether diet and VA associated changes in ECE2 expression translate directly into altered enzymatic activity or peptide-processing capacity. While pre-differentiated SH-SY5Y cells provided valuable cell-autonomous insights, monocultured cell lines cannot fully capture the complex intercellular cross-talk between neurons and glial cells such as astrocytes. This structural simplification may explain the discrepancy observed in ECE2 expression patterns, where acute lipid exposure suppressed ECE2 levels *in vitro*, whereas chronic high-fat diet feeding induced compensatory ECE2 up-regulation in the mice cortex. The lack of ECE2 knockdown/knockout experiments is a major limitation. Therefore, the causal dependence of the observed Aβ effect on ECE2 remains to be established. In addition, the *in vitro* model used PA and RA as pharmacological surrogates and cannot fully replicate the chronic dietary intervention in mice. Extrapolation from SH-SY5Y monocultures to the *in vivo* APP/PS1 cortex should be made with caution. Another limitation is the use of Aβ_25 − 35_ rather than full-length Aβ_40_ or Aβ_42_ in the *in vitro* experiments. Although Aβ_25 − 35_ is a widely used surrogate for Aβ-induced toxicity, it does not fully represent the physiological processing, aggregation, or clearance of endogenous Aβ species. Finally, this study was conducted exclusively in male mice to avoid potential confounding variability driven by estrous cycle hormonal fluctuations. Given the established sex differences in AD progression and estrogen-mediated neuroprotection, our findings may not fully generalize to the females. Future investigations incorporating region-specific analyses, multi-cell co-cultures, and cell-type-resolved methodologies will be essential to further dissect the multicellular networks through which dietary fat and vitamin A status drive Alzheimer's disease pathology.

## Conclusion

High fat, high sugar and/or VA deficiency significantly remodel the gene expression profile in the cerebral cortex of APP/PS1 mice. A diverse set of genes governing Aβ metabolism, neuronal differentiation, myelin integrity, and metabolic signal transduction including *Ece2, Hes3, Fam168b, Prlr, Sstr4*, and *Ucn3* exhibit specific expression profiles that vary according to the lipid and VA nutritional status. Notably, ECE2 appears to be a nutrient-sensitive candidate regulator potentially involved in amyloid processing and Aβ distribution. Our findings identify candidate molecular pathways that may link nutritional factors to AD-related amyloid metabolism, but their functional relevance requires further investigation.

## Data Availability

The datasets presented in this study can be found in online repositories. The names of the repository/repositories and accession number(s) can be found in the article/Supplementary material.
