# Supplementary material for: Impact of high fat and low vitamin A intervention on cortical transcriptional profiles in APP/PS1 mice and neuronal EEF1AKMT4-ECE2-associated amyloid metabolism
Source: Front Nutr. 2026 Oct 22;13:1923538. doi: 10.3389/fnut.2026.1923538 (PMC13736747; doi:10.3389/fnut.2026.1923538)
Supplement: Supplementary file 1 [file Table_1.docx]

**Supplementary Table 1 Components of intervention feed**

| Feed (g/kg) | APP-CON-NVA | APP-CON-LVA | APP-FAT-NVA | APP-FAT-LVA |
| --- | --- | --- | --- | --- |
| casein | 200 | 200 | 200 | 200 |
| cystine | 3 | 3 | 3 | 3 |
| methionine | 3 | 3 | 0 | 0 |
| corn starch | 650 | 650 | 0 | 0 |
| fructose | 0 | 0 | 200 | 200 |
| maltodextrin | 37.5 | 37.5 | 100 | 100 |
| sucrose | 0 | 0 | 54.5 | 54.5 |
| cellulose | 50 | 50 | 50 | 50 |
| palm oil | 0 | 0 | 157.5 | 157.5 |
| corn oil | 50 | 50 | 25 | 25 |
| soybean oil | 0 | 5 | 0 | 0 |
| lard | 0 | 0 | 20 | 20 |
| complex mineral | 35 | 35 | 45 | 45 |
| bitartrate | 2 | 2 | 2 | 2 |
| vitamin D | 1000 IU | 1000 IU | 1000 IU | 1000 IU |
| vitamin A (500000IU/gm) | 4000 IU | 400 IU | 4000 IU | 400 IU |
| vitamin E | 65.1 IU | 65.1 IU | 65.1 IU | 65.1 IU |
| Energy (%) | | | | |
| *Protein* | 20.8 | 20.8 | 20 | 20 |
| *Carbohydrate* | 67.7 | 67.7 | 35 | 35 |
| *Fat* | 11.5 | 11.5 | 45 | 45 |

APP, APP/PS1 mice; CON-NVA, normal fat and fructose + normal vitamin A diet; CON-LVA, normal fat and fructose + low vitamin A diet; FAT-NVA, high fat and fructose + normal VA diet; FAT-LVA, high fat and fructose + low vitamin A diet.

**Supplement Table 2 The function of the selected gene**

| **Genes** | **Function** |
| --- | --- |
| *ECE2* | Limit the accumulation of brain amyloid beta peptides. |
| *EIF4EBP2* | Regulate eIF4E activity, mediate the translation of hormones, growth factors and other regulatory proteins |
| *FAM168B* | Mediate the central nervous system. Its deletion may induce myelin defects by affecting the transcription process. |
| *GP1BB* | Regulate the immune system and is involved in a protein-activated cascade. Associated with delayed AD. |
| *HES3* | Induce insulin release or regulate neural stem cell renewal during development. |
| *IFI27L2A* | Cause neuroinflammation through reactive oxygen species and regulate microglia function and neuroinflammation in the brain. |
| *IGIP* | Associate with blood integrity of insulin in patients with type 2 diabetes. |
| *PHIP* | Induce oxidative impairment of PD-associated brain regions/neurons. |
| *PRLR* | Regulate metabolism, protein transport, signal transduction and cell cycle. |
| *SSTR4* | Associate with the the central nervous system and gastrointestinal tract |
| *TGTP1* | Related to the immune activation function of the body. |
| *UCN3* | Regulate the appetite of mammals by inhibiting food intake and gastrointestinal emptying through the central feeding system. |

**Supplement Table 3 Summary of sequencing data quality (mean** $\boldsymbol{\pm}$**SEM)**

| Group | *n* | Raw reads | Clean reads | Q20 | Q30 | GC pct |
| --- | --- | --- | --- | --- | --- | --- |
| APP-FAT-NVA | 6 | 56628717.7$\pm$6897949.8 | 55301153.0$\pm$6757314.2 | 98.52$\pm$0.06 | 94.76$\pm$0.19 | 49.90$\pm$0.75 |
| APP-FAT-LVA | 6 | 57683754.7$\pm$3891150.6 | 56335718.7$\pm$3790810.1 | 98.51$\pm$0.07 | 94.73$\pm$0.19 | 50.69$\pm$0.22 |
| APP-CON-NVA | 6 | 60327716.7$\pm$5535032.5 | 58881571.0$\pm$5463944.6 | 98.55$\pm$0.09 | 94.84$\pm$0.26 | 50.23$\pm$1.85 |
| APP-CON-LVA | 5 | 56062616.8$\pm$4693403.5 | 54823073.2$\pm$4624855.0 | 98.59$\pm$0.08 | 94.98$\pm$0.23 | 51.05$\pm$0.16 |

Group, group name; Raw reads, total number of reads; Clean reads, Number of filtered reads; Q20, The ratio (%) of total bases exceeding Phred value 20; Q30, The ratio (%) of total bases exceeding Phred value 30; GC pct, GC content (%) in reads after filtration.

**Supplement Table 4 Genomic distribution of sequenced fragments (mean**$\boldsymbol{\pm}$**SEM)**

| Group | *n* | Exon (%) | Intron (%) | Intergenic (%) |
| --- | --- | --- | --- | --- |
| APP-FAT-NVA | 6 | 85.08$\pm$2.23 | 5.70$\pm$0.78 | 9.22$\pm$2.71 |
| APP-FAT-LVA | 6 | 86.53$\pm$0.52 | 6.64$\pm$0.62 | 7.11$\pm$0.81 |
| APP-CON-NVA | 6 | 83.53$\pm$6.71 | 6.15$\pm$0.47 | 10.31$\pm$6.68 |
| APP-CON-LVA | 5 | 86.30$\pm$0.89 | 6.38$\pm$0.35 | 7.32$\pm$0.63 |

Group, group name; Exon (%), reads in the exon region of genome; Intron (%), reads in the intron region of genome; Intergenic (%), reads in the intergenic region.

**Supplement Table 5 Differential expression genes by transcriptomic sequencing**

| Gene | Log_2_ ^Fold-change^ | *p Value* | *q Value* | result |
| --- | --- | --- | --- | --- |
| APP-FAT-LVA vs APP-CON-LVA | | | | |
| *GP1BB* | 2.576006713 | 5.45E-07 | 7.16E-05 | up |
| *IFI27L2A* | 1.025132882 | 0.001192656 | 0.016852231 | up |
| *SSTR4* | -1.042469719 | 1.74E-06 | 0.00016111 | down |
| *HES3* | -1.676292783 | 0.002771157 | 0.030614185 | down |
| *PHIP* | -2.623269005 | 1.81E-07 | 3.43E-05 | down |
| APP-FAT-NVA vs APP-FAT-LVA | | | | |
| *IGIP* | 7.009862386 | 1.49E-07 | 0.002180365 | up |
| *PHIP* | 2.868580569 | 1.15E-07 | 0.002180365 | up |
| *FAM168B* | -1.479621227 | 3.97E-06 | 0.038738234 | down |
| APP-FAT-LVA vs APP-CON-NVA | | | | |
| *IFI27L2A* | 1.224220041 | 2.86E-05 | 0.013466649 | up |
| *TGTP1* | 1.141549917 | 6.83E-07 | 0.000906007 | up |
| *EIF4EBP2* | -1.068195501 | 2.32E-05 | 0.012860684 | down |
| *PHIP* | -3.156362333 | 3.42E-111 | 5.45E-107 | down |
| APP-CON-NVA vs APP-CON-LVA | | | | |
| *PRLR* | 1.766772319 | 3.91E-06 | 0.022566385 | up |
| *UCN3* | 5.707730825 | 7.31E-07 | 0.00527353 | up |
| *ECE2* | 12.97210415 | 5.96E-14 | 1.72E-09 | up |
| APP-FAT-NVA vs APP-CON-NVA | | | | |
| *IGIP* | 6.538453023 | 1.01E-06 | 0.002751285 | up |
| *SSTR4* | -1.235659719 | 1.68E-07 | 0.000839407 | down |
| *ECE2* | -12.97210415 | 1.65E-13 | 3.16E-09 | down |

log_2_FoldChange, log_2_ value of the difference multiple; *p* Value, statistical difference significance test index; *q* Value, indicates the corrected *p* value.
